# Genome-Wide Identification, Expression and Interaction Analysis of *GLN* Gene Family in Soybean

**DOI:** 10.3390/cimb46120847

**Published:** 2024-12-15

**Authors:** Xin Hao, Yiyan Zhang, Hui Zhang, Gang Yang, Zhou Liu, Huiwei Lv, Xiaomei Zhou

**Affiliations:** 1 College of Food Science and Engineering, Boda College of Jilin Normal University, Siping 136000, China; haoxin32857@163.com (X.H.); zh20200409@126.com (H.Z.); young000001@126.com (G.Y.); liuzhou0517@163.com (Z.L.); 18904443011@163.com (H.L.); 2 College of International Education and Exchange, Jilin Agricultural University, Changchun 130118, China; zhangyiyan@jlau.edu.cn

**Keywords:** soybean, *GLN* gene family, seed size, expression pattern

## Abstract

As a globally significant economic crop, the seed size of soybean (*Glycine max* [L.] Merr.) is jointly regulated by internal genetic factors and external environmental signals. This study discovered that the GLN family proteins in soybean are similar to the KIX-PPD-MYC transcriptional repressor complex in Arabidopsis, potentially influencing seed size by regulating the expression of the downstream gene *GIF1*. Additionally, β-1,3-glucanase (βGlu) plays a crucial role in antifungal activity, cell composition, flower development, pollen development, abiotic resistance, seed germination, and maturation in soybean. Through a detailed analysis of the structure, chromosomal localization, phylogenetic relationships, and expression situations in different tissues at different stages of the soybean *GLN* gene family members, this research certifies a theoretical foundation for subsequent research on the biological functions of *GLN* genes in soybean. This research incorporated a comprehensive genomic identification and expression analysis of the *GLN* gene family in soybean. The results indicate that the 109 soybean *GLN* genes are unevenly distributed across soybean chromosomes and exhibit diverse expression patterns in different tissues, suggesting they may have distinct functions in soybean morphogenesis. GO enrichment analysis shows that the *GLN* gene family may participate in a variety of biological activities, cellular components, and molecular biological processes, particularly in catalytic activity, cellular components, and metabolic processes. These findings provide important information for comprehending the role of the *GLN* gene family in soybean and offer potential targets for molecular breeding of soybean.

## 1. Introduction

Soybean (*Glycine max* [L.] Merr.) stands as a paramount economic crop globally, supplying essential edible oils, vegetative proteins, and nutrients for human consumption, in addition to serving as a vital feed source for livestock. The ability of soybean root nodules to fix atmospheric nitrogen in symbiosis with rhizobia bacteria also contributes to a reduction in fertilizer dependency in agriculture. These attributes render soybeans an invaluable asset in addressing Chinese food security and the pursuit of sustainable agricultural practices. To bolster soybean yields, it is crucial to explore and comprehend the regulatory mechanisms behind seed-related traits, which can aid in the development of superior soybean varieties [1]. Research has shown that larger seeds, rich in nutrients, tend to exhibit enhanced germination and development, as well as increased stress tolerance [2].

The determination of seed size is a complicated process governed by both molecular biological and environmental factors working in concert [3]. Soybean genes *GmKIX8* and *BS1*, along with their Arabidopsis counterparts *KIX8/9*, are known to suppress seed weight. They are components of a transcriptional repression complex that includes *PPD1/2* (*BS1*’s homologs) and *MYC3/4*. This complex downregulates the expression of *GIF1*, a gene that influences seed size, leading to the formation of lighter and smaller seeds. It is hypothesized that a similar regulatory network involving PPD, KIX, MYC3/4, and *GIF1* could be operational in soybeans to control seed size [4,5,6]. Elevated expression levels of the Arabidopsis KLU-related gene *GmCYP78A72* in soybeans are associated with an increase in seed size. This implies that *GmCYP78A72* likely contributes to seed enlargement by enhancing cell division, which in turn influences the overall seed weight [7,8]. Simultaneously, *PP2C-1* functions as a phosphatase that enhances brassinosteroid (BR) signaling by removing phosphate groups from transcription factors. It interacts with *GmBZR1*, promoting its dephosphorylation, which in turn upregulates the expression pattern of genes that regulate seed morphology, leading to an increase in the 100-seed weight [9]. Moreover, the expression levels of the gibberellin biosynthesis gene *GA20OX* and *FtsH* genes show a positive correlation with the weight of soybean seeds [10].

Plant β-1,3-glucosidase enzyme [11], classified within the glycosyl hydrolase family 17 (GH17) [12,13,14], is identified as an endoglucanase due to its function in catalyzing the hydrolysis of β-1,3-glucan bonds, particularly the (1→3)-β-glucosyl linkages present in laminarin, resulting in the formation of oligosaccharides consisting of two to six glucose units [11]. This enzyme, referred to as βGlu, plays a crucial role in plant defense mechanisms, notably exhibiting antifungal properties [15]. Beyond its involvement in the defense against fungal pathogens, βGlu is associated with various physiological processes, including responses to cold stress, floral development, cellular division, pollen maturation, seed germination, and the overall maturation of the plant [16,17,18,19,20,21]. In this research, we named the glycosyl hydrolase family in soybean as the *GLN* gene family. As of now, there is a dearth of literature concerning the genomic analysis of the soybean *GLN* gene family. This research employs bioinformatics tools to dissect and catalogue the *GLN* genes across the entirety of the soybean genome. It offers an in-depth examination of their structural composition, chromosomal distribution, evolutionary relationships, and tissue-specific expression profiles. The purpose is to lay the foundation for future studies into the biological roles of these genes in soybean, contributing to a more comprehensive understanding of their functions.

## 2. Materials and Methods

### 2.1. Identification of the GmGLN Gene Family

The genomic data for rice (*Oryza sativa*), soybean (*Glycine max*), and *Arabidopsis thaliana* were sourced from the information commons for rice (https://www.ricedata.cn/gene/, accessed on 10 May 2024), Ensembl Plants database (https://plants.ensembl.org/index.html, accessed on 12 May 2024), and Phytozome databases (https://phytozome.jgi.doe.gov/pz/portal.html, accessed on 12 May 2024). Protein sequences belonging to the *GLN* gene family were extracted from Arabidopsis [22], rice [23], and soybean using both the Ensembl Plants and Phytozome databases (https://phytozome.jgi.doe.gov/pz/portal.html, accessed on 14 May 2024). Employing the *Arabidopsis thaliana* GLN protein sequence as a reference, potential GLN protein sequences from *Glycine max* were detected with an E-value threshold of ≤1 × 10^−5^, employing the blast feature within TBtools-II (version 2.086). CDS sequences of the *GLN* family, which were obtained from NCBI (https://www.ncbi.nlm.nih.gov/Structure/bwrpsb/bwrpsb.cgi, accessed on 14 May 2024), were utilized to precisely identify the conserved structural domains existing within these candidate genes. The shortlisted protein candidates, designated as GmGLN1-109, were categorized within the GLN-specific X8 superfamily and the broader Glycohydro superfamily.

### 2.2. Phylogenetic Relationship Analysis

Sequence alignments of GLN proteins from *Arabidopsis thaliana*, *Oryza sativa*, and *Glycine max* were conducted using Clustal W, followed by neighbor-joining clustering analysis (with a bootstrap value of 1000) using MEGAX version 10.2.6 [24]. tvBOT was used to modify and annotate the phylogenetic tree [25]. Subsequently, the genetic relationship was visually enhanced using the ChiPlot online platform (https://www.chiplot.online/, accessed on 14 May 2024).

### 2.3. Gene Structure and Conserved Motif Analysis

The structure of exon–intron for *GmGLN* was identified utilizing the Gene Structure Display Server (https://gsds.gao-lab.org/Gsds_help.php#level1, accessed 14 May 2024) [26]. The conserved motifs of GmGLNs were predicted by utilizing MEME (Multiple Expectation Maximization for Motif Elicitation) (https://meme-suite.org/meme/tools/meme, accessed 28 June 2024), with parameters set as follows: whole motifs, minimum amino acid length of 1, and maximum amino acid length of 50 [27]. Lastly, TBtools-II (version 2.086) was employed to summarize and refine the data.

### 2.4. Chromosome Location

To ascertain the chromosomal positions of *GmGLNs*, we employed the GTF/GFF functionality within TBtools-II (version 2.086) to import the soybean genome annotation files into the Gene Location Visualizer for analysis.

### 2.5. GO Enrichment Analysis

We annotated the GmGLN protein with gene ontology (GO) functional categories utilizing the functional annotation options from the DAVID database (https://david.ncifcrf.gov/, accessed 14 May 2024) [28]. Following the transformation of *p*-values using the −log10 method, the data were visualized as bubble figures utilizing the Omicshare platform (https://www.omicshare.com/tools/Home/Soft/gogseasenior, accessed 14 May 2024).

### 2.6. Expression Pattern Analysis

Transcriptome data, represented by fragments per kilobase of transcript per million mapped reads (FPKM) values, for *GmGLNs* across 61 different tissues of *Glycine max* Wm82.a2. V4 were obtained from the database from Phytozome [29]. These tissues encompassed a range of developmental stages for roots, stems, flowers, leaf buds, pod seeds, cotyledons, shoot meristems, pods, and seed coat layers. The FPKM values, which measure the transcript abundance relative to the total number of reads, were used to assess the expression levels of *GmGLNs*. This metric provides a standardized measure of gene expression by accounting for both transcript length and sequencing depth.

## 3. Results

### 3.1. Construction of Phylogenetic Trees for GmGLN Gene Family

**Figure 1:**
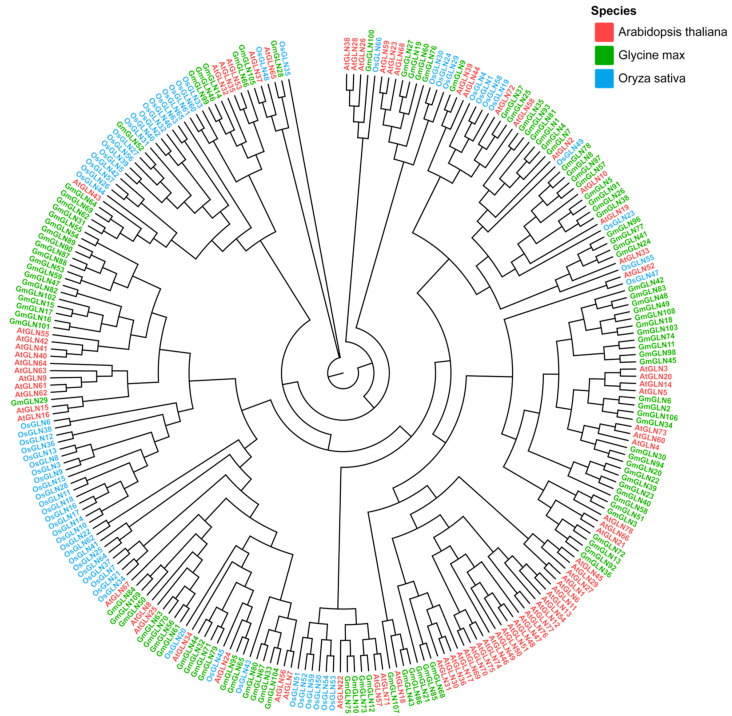
Phylogenetic tree of the *GLN* gene family in *Arabidopsis thaliana*, *Oryza sativa*, and *Glycine max*. A phylogenetic tree was built by using MEGAX (1 × 10^3^ bootstrap replicates). Each species is represented by a distinct color in the phylogenetic tree to facilitate differentiation. Genes prefixed with “Os” prefix designates genes from *Oryza sativa*, “At” prefix indicates genes from *Arabidopsis thaliana*, and “Gm” corresponds to *Glycine max*.

We generated a phylogenetic tree utilizing the complete protein sequences of the soybean GLN family, as depicted in Figure 1 and Table S1. On the tree, the 109 soybean GLN family proteins are highlighted in green, 66 rice GLN family proteins in blue, and 78 Arabidopsis GLN family proteins in red. Given that the GLN gene family in *Arabidopsis thaliana* has not been systematically classified and studied, a similar lack of classification exists for the soybean GLN family. Consequently, we have redesignated the soybean GLN family genes. The phylogenetic analysis reveals that proteins with tighter clustering exhibit highly similar structures, whereas those with more distant clustering display significant structural variations. This suggests that distinct GLN family members may have experienced divergent evolutionary trajectories.

### 3.2. Gene Conserved Motif Analysis of the GmGLN Gene Family

**Figure 2:**
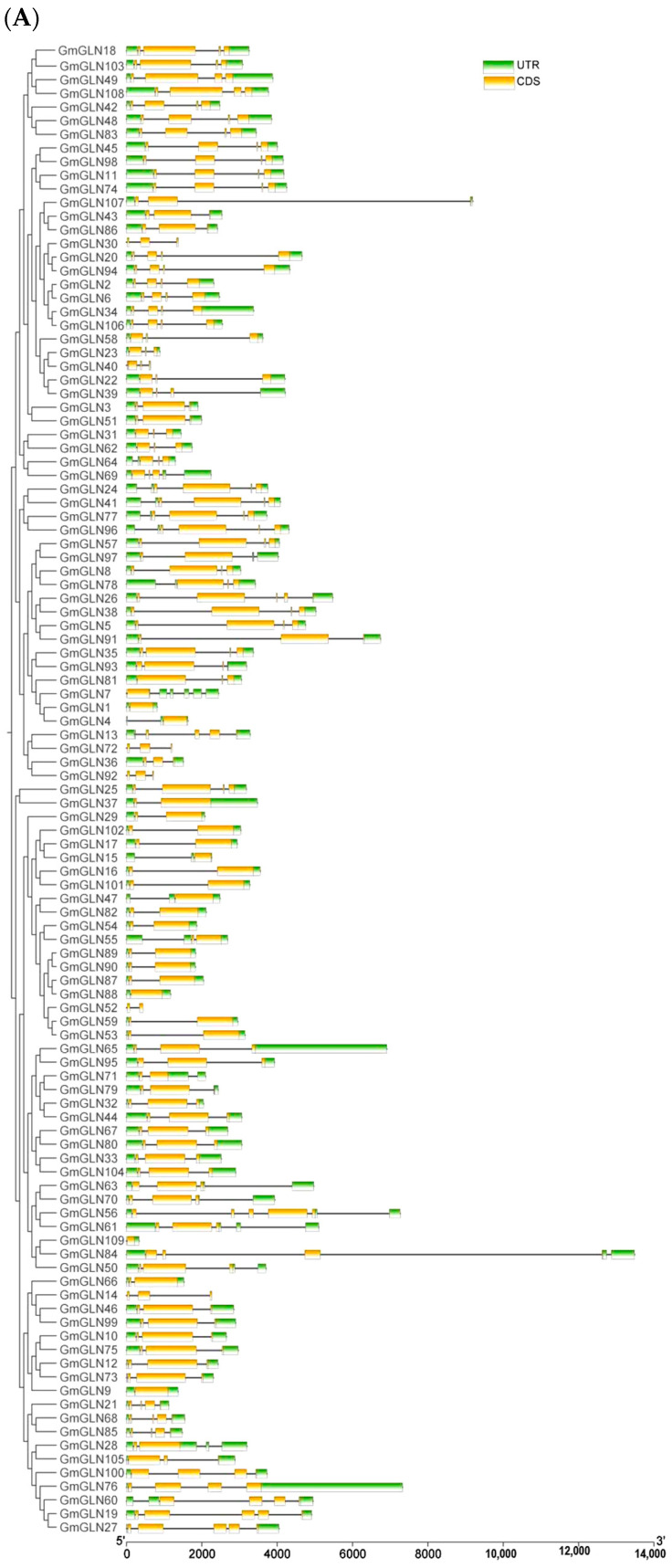

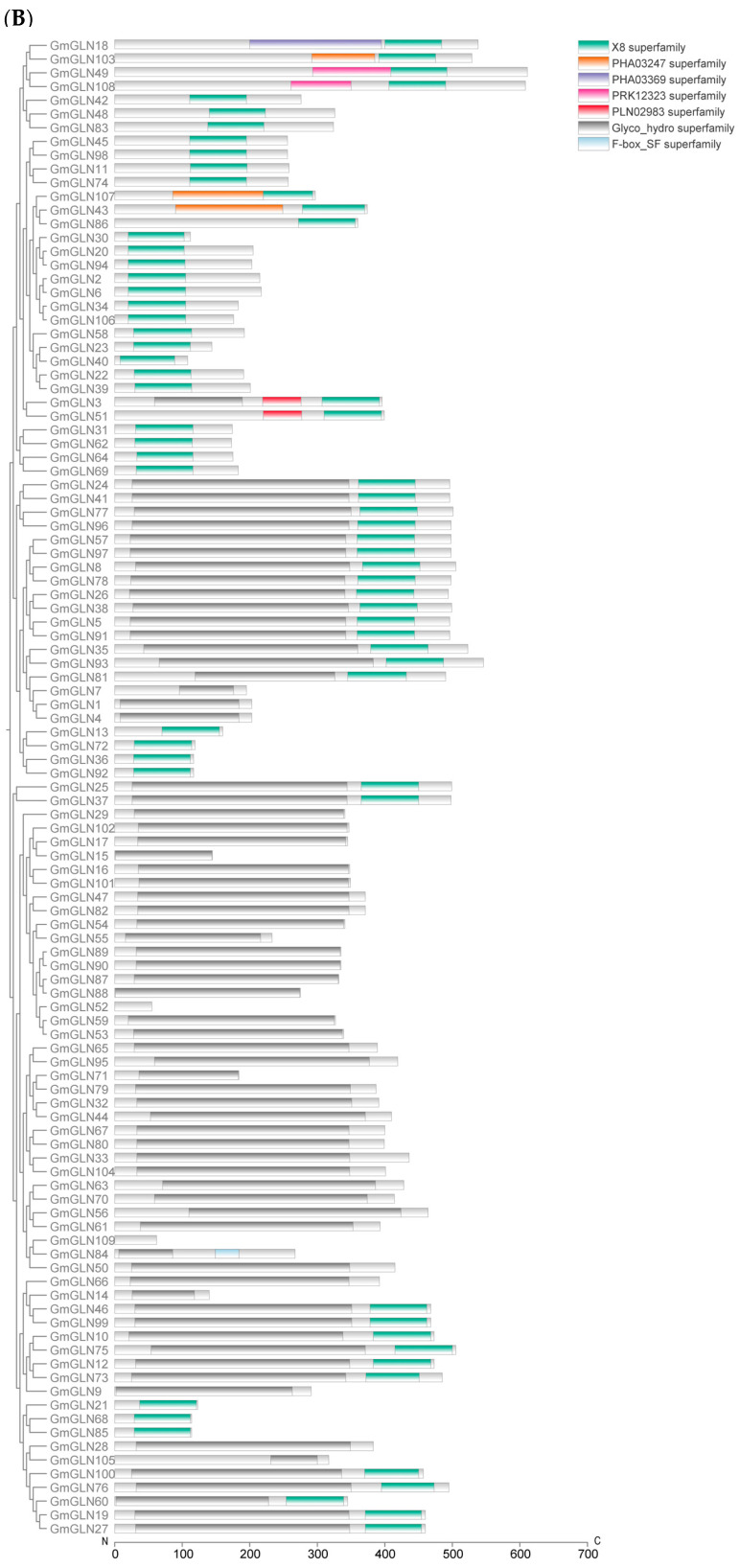

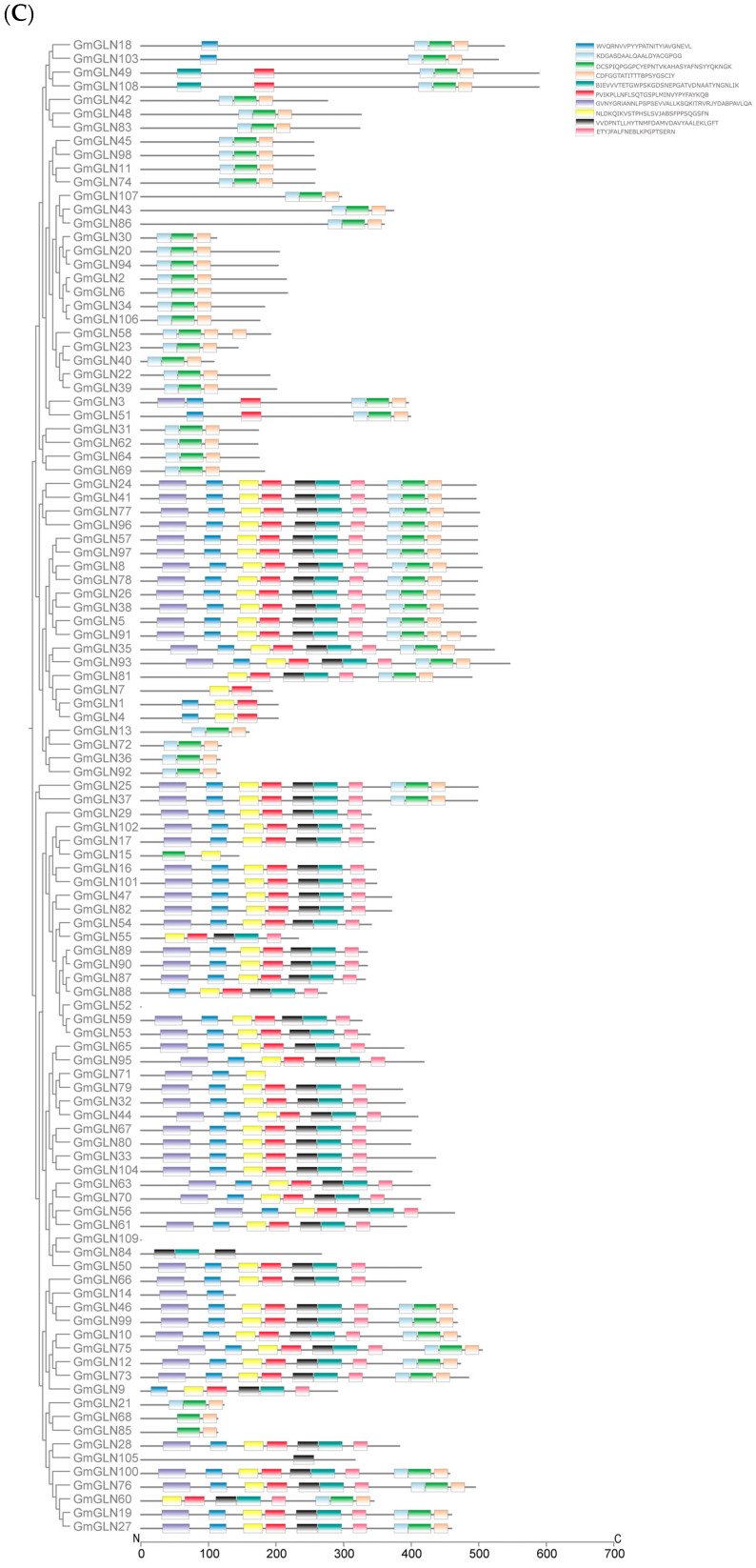
Analysis and prediction of gene structure, conserved motifs and domains for *GmGLN* genes. (**A**) Gene structure of *GmGLN* genes—green boxes indicate 5′or 3′ UTR regions, yellow boxes indicate exons, and lines with black represent introns. (**B**) Conserved domains for *GmGLN* protein family. (**C**) Conserved motifs for *GmGLN* protein family.

To the structural diversity or evolutionary patterns of the *GmGLN* genes, we first constructed a phylogenetic tree and then proceeded to analyze their gene structures, conserved domains, and motifs. The gene structure analysis indicated that all *GmGLN* genes are composed of multiple introns and exons, as presented in Figure 2A, with the number of exons ranging from a single exon in *GmGLN15* to five in *GmGLN19*. Among the genes examined, 109 *GmGLN* genes included an untranslated region (UTR), and genes within the same group displayed similar gene structures, suggesting a conserved exon–intron architecture among the *GmGLN* genes. Employing TBtools, we identified conserved structural domains within the *GmGLN* genes, finding that all *GmGLN* genes contained GLN-specific domains, such as the X8 superfamily and Glycohydro superfamily domains, as illustrated in Figure 2B.

Additionally, individual genes harbored other conserved domains, potentially linked to their specific gene functions. The website (MEME) was employed to predict the fifteen conserved motifs (Motif1-15) for GmGLN protein in Figure 2C. Our research disclosed that 24 GmGLN protein harbored the complete set of conserved motifs, and approximately half of the genes analyzed featured either Motif1 or Motif2, suggesting their suitability as identifiers for GLN proteins. The joint phylogenetic tree analysis demonstrated that genes within the same subfamily showed congruence in the quantity and arrangement of motifs. On the other hand, there was a notable divergence in motif distribution among genes from separate subfamilies, potentially reflecting their functional differences.

### 3.3. Chromosome Location Analysis

**Figure 3:**
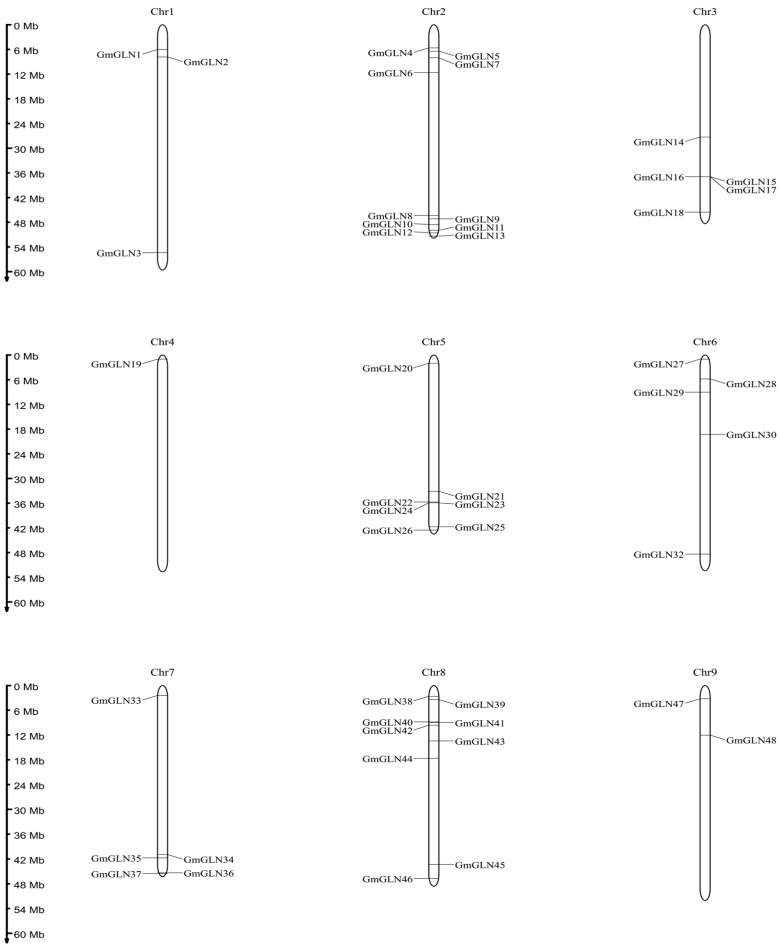

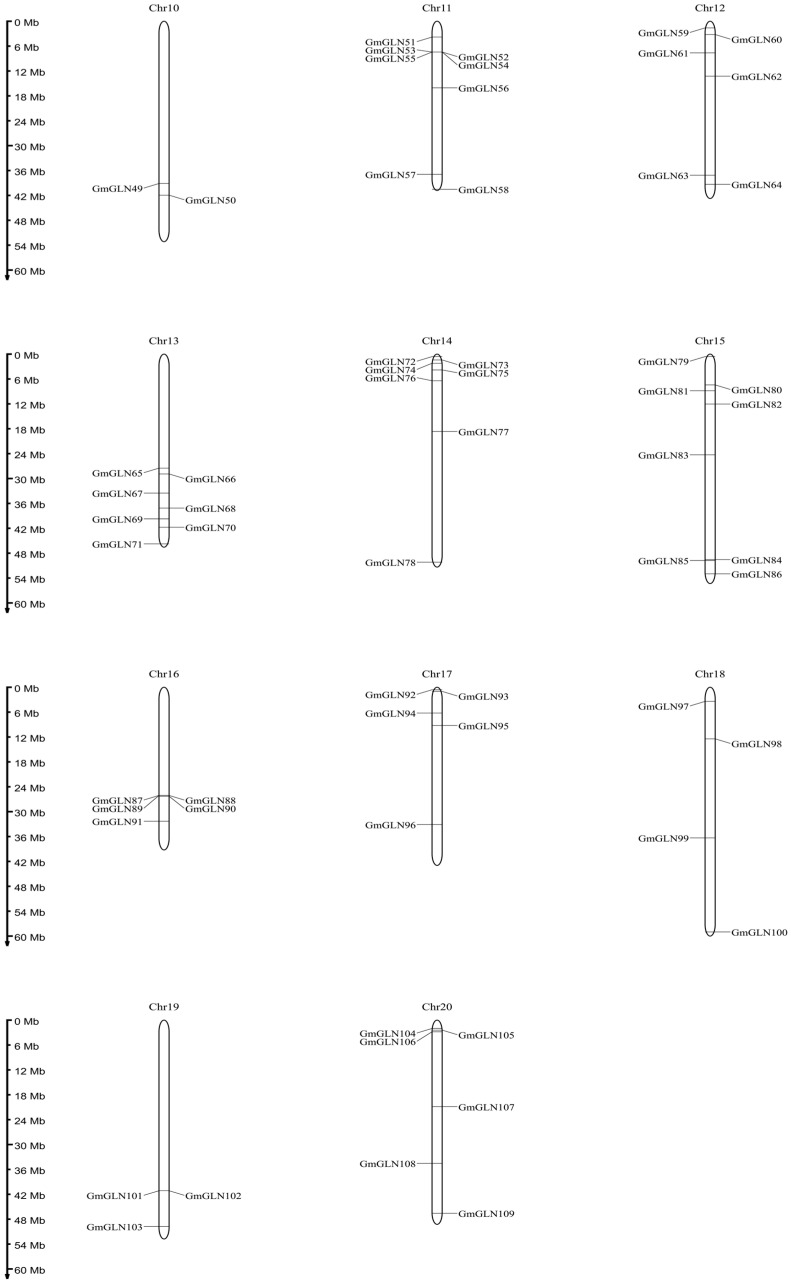
Localization of *GmGLN* genes on chromosomes. A total of 109 *GmGLN* genes are distributed across chromosomes 1 through 20. Each vertical bar is annotated with the corresponding chromosome number at its apex. A scale indicating the chromosome length in megabases (Mb) is provided alongside.

Utilizing the annotation of the soybean genome, we delineated the chromosomal locations of the *GmGLN* genes, which highlighted a non-uniform distribution of the 109 *GmGLN* genes across the 20 chromosomes, as depicted in Figure 3.

### 3.4. Analysis for Gene Ontology (GO)

**Figure 4:**
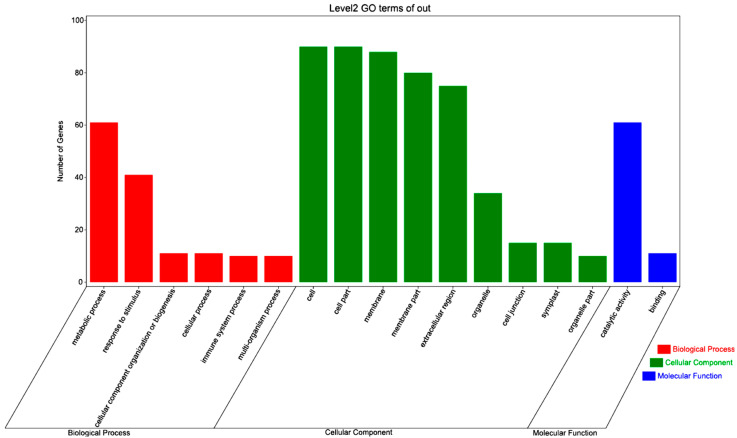
Analysis of gene ontology (GO) enrichment for the *GmGLN* gene family was stratified into three principal domains: biological process (BP), cellular component (CC), and molecular function (MF). GO terms with a *p*-value threshold of less than 5 × 10^−2^ were considered to be statistically significant.

The enrichment analysis for gene ontology (GO), a prevalent approach for deciphering genes for metabolic pathways they may be involved in, was applied to the *GmGLN* gene family. Our findings indicate that these genes are likely engaged in a variety of biological, cellular, and molecular processes, encompassing six biological process categories, nine cellular component categories, and two molecular function categories, as depicted in Figure 4. The prevalent term within the molecular function category was “catalytic activity,” hinting at the diverse catalytic roles that *GmGLN* genes might play in the modulation of molecular pathways. The “cell part” category was the most significantly enriched in cellular components, while the “metabolic process” category stood out in biological processes. Collectively, these results imply that *GmGLN* genes potentially act as enzymes within cellular structures, participating in both metabolic and catalytic activities.

### 3.5. Expression Status of GmGLN Gene Family for Soybean in Various Tissues

**Figure 5:**
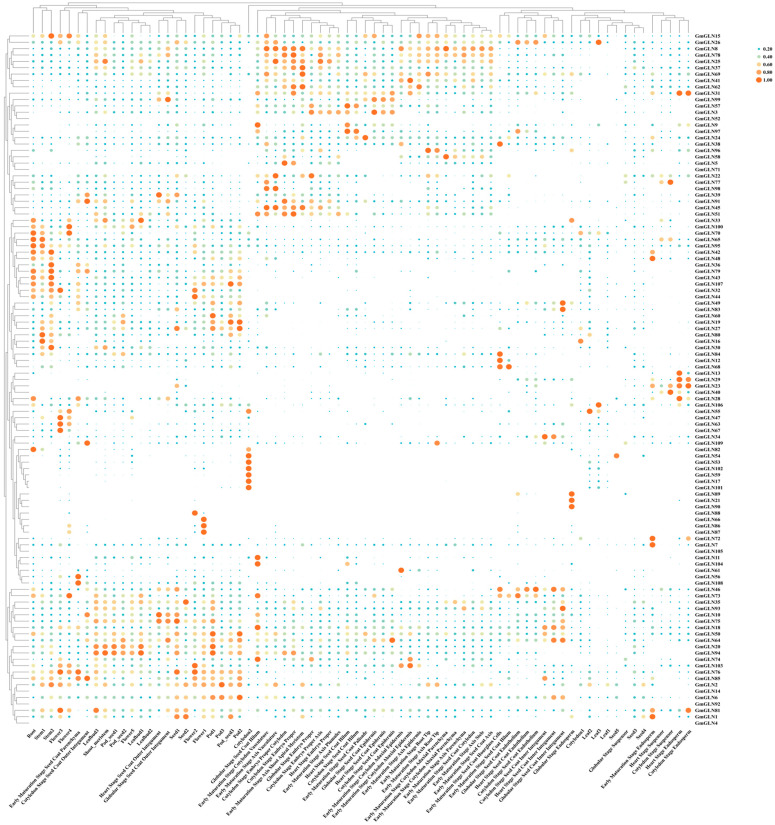
Heat map of the *GmGLN* gene family in different tissues for soybean. The heat map utilizes a color scale on its right side to represent the relative expression level, with a gradient ranging from light green to orange-red indicative of a progressive increase in expression.

In order to certify the roles of *GmGLN* genes in the developmental processes of *Glycine max*, we conducted an analysis of the expression profiles for each gene within the *GmGLN* family. By extracting gene expression data from the Phytozome genome database, we assessed the transcriptional activity across a range of soybean tissues. These tissues included roots, stems, flowers, leaf buds, pod seeds, cotyledons, shoot meristems, pods, and seed coat layers throughout their developmental stages, as illustrated in Figure 5. The expression of *GmGLN55* is most prominent in leaf2. Conversely, all *GmGLN* genes exhibited lower expression levels in leaf1. *GmGLN66*, *GmGLN88*, *GmGLN86*, and *GmGLN87* showed increased expression specifically in flowers. *GmGLN65* and *GmGLN82* exhibited higher expression levels in roots and cotyledon but were lowly expressed in other tissues. *GmGLN54*, *GmGLN53*, *GmGLN102*, *GmGLN59*, and *GmGLN17* displayed high expression levels specifically in cotyledon2 and moderate expression in other tissues. A majority of *GmGLN* genes exhibited low expression levels in leaves. Based on these findings, we posit that individual *GmGLN* genes may contribute uniquely to soybean development, while collectively, they appear to play pivotal roles in stem and flower development.

## 4. Discussion

### 4.1. Soybean Seed Size QTLs

Soybean seed dimensions constitute a pivotal domestication characteristic that significantly contributes to high soybean yields. Nevertheless, the genetic underpinnings of the variation in seed size within soybean populations remain largely unexplored. Presently, SoyBase documents 398 quantitative trait loci (QTLs) associated with seed weight, with 94 of these QTLs having been pinpointed through genome-wide association studies (GWAS) (https://www.soybase.org/ (accessed on 16 May 2024)). Within this set, 26 QTLs are mapped to chromosome 11. The identification of the genes responsible for these QTLs presents a significant challenge, primarily due to the absence of suitable populations for genetic mapping. The establishment of primary mapping populations is essential for the mapping of QTLs [30].

### 4.2. β-1,3-Glucosidase Regulates Soybean Seed Size

The β-1,3-glucosidase enzyme (βGlu), which includes an N-terminal sequence (NTS) and belongs to the glycoside hydrolase family 17 (GH-17) domain, is commonly linked to a range of plant processes such as antifungal responses, cold tolerance, floral development, cellular division, pollen maturation, seed germination, and maturation [15,16,17,18,19,31]. In soybeans, the *HSW* gene encodes a full-length β-Glu homolog that is implicated in analogous functions, maintaining the standard glucosidase activity. A 14bp deletion in the *HSW* gene results in the hsw variant, which introduces a frameshift and premature stop codon in certain soybean lines. The *HSW* allele is associated with significant alterations in seed and cell size in both wild and cultivated soybeans, suggesting a possible role for hsw in the regulation of seed size [15]. This study reveals that the *GLN* family genes are highly expressed in seed tissues, suggesting their involvement in seed development. In Arabidopsis, the *GL7* gene, a member of the *GLN* family on chromosome 7, is a key QTL for grain size. Plants with the *GL7* gene show elongated cells, narrower cell widths, an increased grain length-to-width ratio, and larger, more compact starch granules, enhancing grain appearance [32]. In rice, the *GL7* gene also plays a role in determining grain size and shape, indicating a conserved function of *GLN* genes across species. This function may include regulating growth and development and responding to environmental stresses, especially regarding nitrogen use efficiency [33,34]. In Arabidopsis, the absence of *GLN1;1, GLN1;2*, and *GLN1;3* genes leads to reduced vegetative growth and seed size, highlighting the importance of *GLN* genes in seed size regulation [35]. For soybeans, the patterns of expression and functions of the *GmGLN* gene family are likely directly correlated to seed development and size, which are key determinants of soybean yield and quality [36].

GO analysis also reveals that the products of soybean *GLN* family gene expression have enzymatic activity and are involved in cell components and development. This suggests that the *GLN* genes are instrumental in cellular metabolism and developmental processes, which may have a bearing on the cell structure and functionality in soybeans, ultimately impacting the growth and development of the entire plant.

In addition, this study found that the soybean *GLN* family genes have high similarity in conserved domains and high homology. Moreover, the distribution of the *GLN* family genes on the soybean chromosomes is not uniform, with ten *GLN* genes located on four chromosomes (4, 7, 9, 10), and more *GLN* family genes located on four other chromosomes (2, 8, 11, 13–15). Chromosome 11 has been found to contain *GLN* genes that can regulate seed size [36], suggesting that other *GLN* genes on chromosome 11 may also have this function. In-depth study of other *GLN* family genes on chromosome 11 could provide a deeper understanding of seed development.

## 5. Conclusions

In our research, we discovered a total of 109 *GmGLN* genes from the soybean genome. We performed a comprehensive analysis to explore the phylogenetic ties, gene architecture, conserved domain structures, and motif compositions of these *GLN* genes, which aids in understanding their evolutionary connections with other plants. The gene expression data from different soybean tissues demonstrated the varied expression status characteristic of the *GLN* gene family. Furthermore, our speculation about the functions of the GmGLN genes indicates that they may play a significant role in regulating plant growth and development. These discoveries lay a solid groundwork for subsequent studies focused on deciphering the functional aspects of *GLN* genes.

## Data Availability

Data are contained within the article and Supplementary Materials.

## Supplementary Materials

The following supporting information can be downloaded at: https://www.mdpi.com/article/10.3390/cimb46120847/s1.
